# Re-evaluation of the species of hookworms infecting dogs in Central Vietnam

**DOI:** 10.1186/s13071-015-1015-y

**Published:** 2015-07-28

**Authors:** Dinh Ng-Nguyen, Sze Fui Hii, Van-Anh T Nguyen, Trong Van Nguyen, Dien Van Nguyen, Rebecca J Traub

**Affiliations:** Faculty of Animal Science and Veterinary Medicine, Tay Nguyen University, Buon Ma Thuot, Dak Lak Vietnam; Faculty of Veterinary and Agricultural Sciences, University of Melbourne, Parkville, VIC 3052 Australia; School of Veterinary Science, The University of Queensland, Gatton, 4343 Australia

**Keywords:** Hookworm, Dog, Zoonosis, Ancylostoma

## Abstract

**Background:**

Differentiation of canine hookworm species is crucial from both a veterinary and public health standpoint. In Vietnam, three hookworm species, namely *Ancylostoma caninum, Ancylostoma braziliense* and *Uncinaria stenocephala* are reported to infect dogs. In light of the emerging distribution of *A. ceylanicum* in Asia, this study aims to re-evaluate the status of *Ancylostoma* in dogs in Vietnam.

**Methods:**

Faecal samples collected from 200 community dogs in Dak Lak province were subjected to faecal floatation for the detection of hookworm eggs. Hookworm-positive samples were subjected to a PCR-Restriction Fragment Length Polymorphism (PCR-RFLP) assay targeting the internal transcribed spacer (ITS) region of rDNA for hookworm species identification. A subset of hookworm-positive samples was also subject to haplotype characterisation at the cytochrome oxidase-1 (COX-1) gene. Detailed morphological criteria were utilised in addition to molecular markers, to identify adult hookworms recovered from necropsied dogs.

**Results:**

Of 200 canine faecal samples, 111 (55.5 %) were positive for hookworm eggs on faecal flotation. Of these, 94/111 (84.7 %) were successfully amplified and assigned species status by PCR-RFLP targeting the ITS region. In total, 54.3 % (51/94) dogs harboured single infections with *A. ceylanicum*, 33.0 % (31/94) with *A. caninum,* and 12.7 % (12/94) harboured mixed infections with both *A. ceylanicum* and *A. caninum*. Adult worms recovered from necropsied dogs matched morphological description provided for *A. ceylanicum*, Looss (1911) for which the mediolateral and posteriolateral rays are parallel. Characterisation of the COX-1 gene placed all Vietnamese canine isolates of *A. ceylanicum* within the ‘zoonotic’ haplotype.

**Conclusion:**

Based on this information, it is apparent that the hookworms present in dogs in Vietnam are those of *A. ceylanicum* and not *A. braziliense*. Owing to the endemic nature of this significant zoonosis in dogs, the study strongly advocates for specific identification of this hookworm in human hookworm surveys.

## Background

*Ancylostoma caninum, Ancylostoma braziliense* and *Ancylostoma ceylanicum* are ubiquitous hookworms of dogs in the tropics, whereas the distribution of *Uncinaria stenocephala* is limited to regions and territories with cool and temperate climates [1, 2]. The principal veterinary importance of these hookworms arises from their ability to cause anaemia and hypoproteinemia in the canine host. From a morbidity standpoint, the most important difference between the three species is that *A. caninum* causes far greater blood loss per worm (0.08 - 0.2 ml/day) [3] than *A. ceylanicum* (0.033 ml/day) [4], *A. braziliense* (0.002 ml/day) and *U. stenocephala* (0.0003 ml/day) [3]. All canine hookworms are zoonotic and capable of producing cutaneous larva migrans or ‘ground itch’ in humans, however, *A. braziliense* is the only species responsible for producing prolonged ‘creeping eruptions’, with tracts in the skin being recorded for over 100 days in some cases [5]. In addition to this, the pre-adult stage of *A. caninum* is a well-recognised aetiological agent of eosinophilic enteritis [6]. *Ancylostoma ceylanicum* is the only species of canine hookworm capable of producing natural patent infections in humans, with accompanying eosinophilic enteritis and anaemia [7, 8]. The parasite is the second most common hookworm of humans in Southeast Asia, estimated to infect between 70–100 million people in areas where the hookworm is endemic in animals [9, 1]. *A. ceylanicum* infection has been reported infecting dogs, cats and/or humans in almost all regions of the Asia Pacific and recently Myanmar [10], except for Vietnam.

In Vietnam, Houdemer [11] was the first to identify the presence of *A. caninum* and *A. braziliense* in dogs by morphological identification of adult worms in the central city of Hue and southern city of Ho Chi Minh. In Hanoi, both *A. caninum* and *U. stenocephala* in dogs were reported at a prevalence of 57.7 % and 12.7 %, respectively [12]. In Ho Chi Minh city, *A. caninum, A. brazilience*, and *U. stenocephara* were reported in dogs at prevalence rates of 79.84 %, 11.06 % and 63.60 %, respectively [13], while in Central Vietnam the prevalence of these hookworm species were reported at 49.61 %, 25.15 % and 30.06 %, respectively [14].

During the first half of the 20th century, *A. ceylanicum* was regarded as a synonym of *A. braziliense* [15] until Biocca [16] provided morphological evidence that they were different. In light of the emerging knowledge of the distribution of *A. ceylanicum* in Asia, this study re-evaluates the previous reports of the presence of *A. braziliense* in Vietnam by utilising both conventional and molecular tools to identify hookworm species in stray dogs located in Dak Lak province in the Central Highlands of Vietnam.

## Methods

### Study site and sampling

The study was conducted in February 2014 in Dak Lak province located in the Central Highlands of Vietnam (12° 40’ 0” N, 108° 3’ 0” E). Dak Lak lies 500 m above sea level and experiences a tropical climate with rainy and dry seasons [17] with an average annual rainfall of 1900 mm [18].

Households were randomly chosen from six rural villages surrounding Buon Ma Thuot, in Dak Lak province. Following verbal consent from owners, single faecal samples were collected from the rectum or freshly off the ground following defecation from 200 community dogs. Dogs ranged in age from 6 weeks to 6 years. Dogs in these communities are semi-domesticated and allowed to roam freely and likely to share environments with other animals, including cats. They have limited or no access to veterinary attention or deworming. Faecal samples were stored in a cool box and transferred to Tay Nguyen University the same day for parasitological examination. In a separate tube, 5 g of each faecal sample were fixed in 5 % potassium dichromate (w/v) and exported to the University of Melbourne for molecular analysis. Necropsies and intestinal washings were performed on ten cadavers of owner-donated dogs that died due to gastroenteritis at the Tay Nguyen University Veterinary Teaching Hospital. Briefly, the small intestines were sectioned, cut open, the mucosa scraped and washed into a tray. Intestinal contents were then filtered through a metal sieve (500-μm aperture) and adult hookworms were isolated and preserved in a 2 ml tube containing 75 % alcohol.

The Scientific Committee of the Faculty of Animal Science and Veterinary Medicine (FAVM), Tay Nguyen University (reference number 30/K.CNTY), provided animal ethics approval for this project.

### Parasitological methods

At the laboratory, 2 g of each faecal sample was subjected to a faecal flotation using saturated sodium nitrate solution S.G. 1.20 [19] and the presence of hookworms eggs noted.

The heads and tails of randomly selected adult hookworms sourced from donated dog cadavers were removed and cleared in lactophenol and placed on slides for microscopic examination, while the bodies were placed back in 70 % ethanol for identification by PCR. Male specimens possessing a single pair of teeth were subject to detailed bursal ray morphology. The most reliable difference between *A. braziliense* and *A. ceylanicum* is the configuration of the lateral bursal rays of the male [16, 20]. For *A. braziliense*, all three lateral rays are widely separated at their tips, whereas for *A. ceylanicum*, the mediolateral and posteriolateral are essentially parallel*,* so that the tips of these rays are very close together, but separated from the tip of the externolateral ray.

### Molecular methods

#### DNA extraction

The PowerSoil DNA Isolation Kit® (Mo Bio, Carlsbad, CA, USA) was used to extract genomic DNA from microscopy-positive hookworm faecal samples. The protocol of extraction was in accordance with the manufacturer’s instructions, with the exception that provided beads were replaced with 1 g of Silica/Zirconia 0.5 mm beads (Daintree Scientific, Tasmania, Australia). Final elution of DNA was made in 50 μL of elution buffer. The extracted DNA was stored at −20 °C until amplification.

Bodies of adult hookworms were extracted using the DNeasy Blood & Tissue Kit® (Qiagen, Hilden, Germany) according to the manufacturer’s instructions and eluted in 200 μl of AE Buffer.

### PCR – Restriction Fragment Length Polymorphism (RFLP)

A PCR- RFLP targeting the internal transcribed spacer region (ITS) of ribosomal DNA of *Ancylostoma* and *Uncincaria* was utilised to identify hookworm species [2, 21]. In brief, RTGHFI and RTABCR1 primers were used to amplify a 545-bp region of ITS-1, 5.8S, and ITS-2 of *A. caninum*, *A. ceylanicum*, and *U. stenocephala* hookworms. In a separate PCR, a 673-bp region of *A. braziliense* was amplified by using RTGHF1 and a specific reverse primer, RTAYR1. The PCR assay comprised 10 × CoralLoad PCR Buffer (Qiagen), 12.5 pmol of each primer, 0.5 U of HotStar Taq DNA Polymerase and 2 μL of DNA, in a total volume of 25 μl. The cycling conditions were the same as the published protocol [21] except for an initial denaturation of 5 min at 95 °C. Amplified PCR products (10 μL; RTGHF1/RTABCR1) were digested with *HinF*I and *Rsa*I endonucleases in separate reactions at 37 °C overnight. The RFLP patterns generated by each sample were compared to the expected RFLP profiles for each hookworm species.

### Haplotype characterisation of *A. ceylanicum* at the cytochrome oxidase −1 (COX-1) gene

Ten randomly selected gDNA from faecal samples identified as positive for *A. ceylanicum* on the PCR-RFLP were further subjected to haplotype level analysis of the mitochondrial COX-1 gene using a previously published PCR using primers AceyCOX1F (5′-GCTTTTGGTATTGTA-AGACAG-3′) and AceyCOX1R (5′- CTAACAACATAATAAG-TATCATG-3′) [9].

### DNA sequencing and phylogenetic analysis of COX-1 gene sequences

For adult worms and COX-1 PCRs, products were purified by Exonuclease I (ExoI) and FastAPTM Thermosensitive Alkaline Phosphatase and submitted for bidirectional DNA sequencing at The Melbourne Translational Genomics Platform (The University of Melbourne) or Macrogen Inc (Korea).

DNA sequences resulting from a 377-base pair fragment of the COX1 gene were read with Finch TV version 1.4.0 trace viewer (Geospiza, Inc., Seattle, WA, USA) and aligned with BioEdit version 7.2.3 (www.mbio.ncsu.edu/BioEdit/bioedit.html) using COX-1 gene sequences from the following hookworm species: *A. ceylanicum* Malaysia isolates (GenBank no. KC247727– KC247745, Human Sg Bumbun 19, Human Pos Iskandar 11; Cambodian Isolates (Genbank no. KF896596–KF896605), Southern China isolates (KP072074 and KP072071), *A. caninum* (NC012309) and *A. duodenale* (NC003415.1). Neighbor-joining analyses were conducted by using Tamura-Nei parameter distance estimates on a 257 base pair region of aligned sequences. Bootstrap analyses were conducted using 1,000 replicates and the tree was constructed by using Mega 6 (http://www.megasoftware.net/) [22].

### Statistical analyses

The data were analysed using IBM SPSS Statistics v21 (SPSS Inc., Chicago, IL, USA). The prevalence of hookworm infection was calculated by using descriptive statistics.

## Results and discussion

### Prevalence of canine hookworm species in dogs

**Fig. 1:**
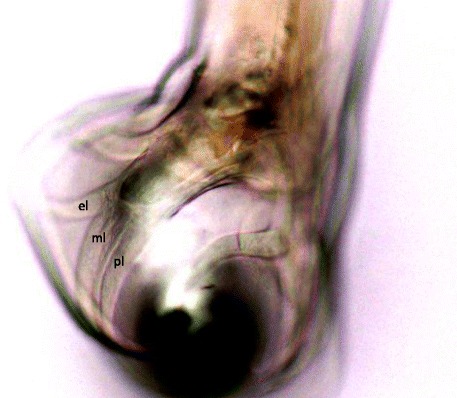
Bursa of male *Ancylostoma ceylanicum* isolated from a dog in Vietnam. Lateral rays are marked as externolateral (el), mediolateral (ml) and posteriolateral (pl)

Copulatory bursae of two male hookworms isolated from necropsied dogs in Dak Lak conformed to the typical morphology of *A. ceylanicum*, in having the mediolateral and posteriolateral rays running parallel to their tips and the externodorsal ray attached relatively distally along the dorsal trunk (Fig. 1). ITS2 sequence data of these two adult male hookworms were 100 % identical to an isolate of *A. ceylanicum* recovered from a dog in northern India (Genbank accession no. DQ780009). This study represents the first report of *A. ceylanicum* in Vietnam. It is likely that the original species designation of *A. braziliense* by Houdemer [11] was erroneous, owing to confusion surrounding their status as a single entity in the early nineteenth century.

Of 200 canine faecal samples, 111 (55.5 %) were positive for hookworm eggs on faecal flotation. Of these, 94/111 (84.7 %) were successfully amplified and assigned species status by PCR-RFLP targeting the ITS region. In total, 54.3 % (51/94) dogs harboured single infections with *A. ceylanicum*, 33.0 % (31/94) with *A. caninum,* and 12.7 % (12/94) harboured mixed infections with both *A. ceylanicum* and *A. caninum*. To date, canine hookworm surveys in Vietnam have relied on morphometric analyses of hookworm eggs according to Monnig [23] or morphological analyses of adult worms, based solely on the numbers of pairs of teeth within the stoma, for the designation of species. Both *A. braziliense* and *A. ceylanicum* possess a single pair of teeth in their buccal capsule and Yoshida [20] noted that using the shape of the teeth to differentiate the two species was difficult. Although eggs of *A. caninum*, *U. stenocephala* and *A. braziliense* can be morphologically distinguished by their size [24], this has yet to be proven for the eggs of *A. ceylanicum*. To date, the distribution of *A. braziliense* in Asia appears to be restricted to the south, below latitude 10 °N. The hookworm has been reported in Malaysia [17, 25], Borneo [26] and Indonesia [27]. Surprisingly, no *U. stenocephala* were identified in the present study despite previous reports of this hookworm in the central and southern regions of Vietnam. We are unable to confirm whether these reports are erroneous, however, future surveys adopting molecular diagnostic methods in conjunction with detailed morphological identification of adults and eggs from the faeces of dogs, will help elucidate this finding.

**Fig. 2:**
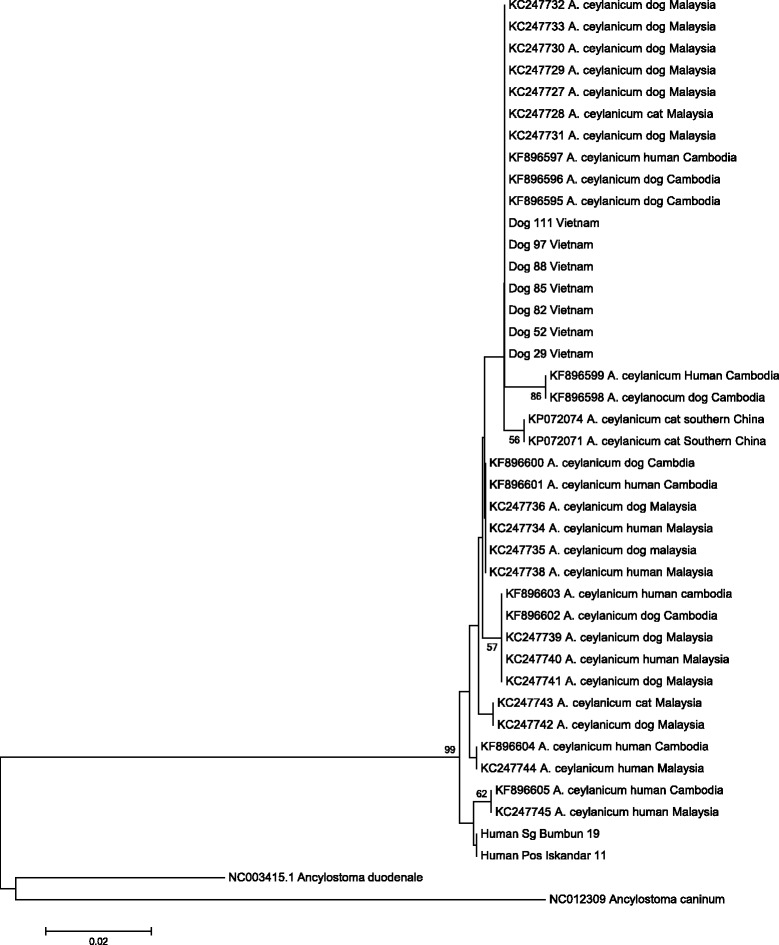
The evolutionary relationship for the COX-1 gene of *A. ceylanicum* inferred using the Neighbour-Joining method. The evolutionary distances were computed using the Maximum Composite Likelihood method and are in the units of the number of base substitutions per site. There were a total of 257 positions in the final dataset. Evolutionary analyses were conducted in MEGA6

Molecular epidemiologic data gathered from characterization of the COX-1 gene of *A. ceylanicum* have divided isolates into two genetically distinct COX-1 haplotypes, the first comprising isolates from humans, dogs, and cats (zoonotic) and the other specific to humans [9, 28]. Out of the ten isolates amplified at the COX-1 gene, seven isolates provided clear and readable sequences for phylogenetic analysis. All seven *Ancylostoma ceylanicum* isolates from dogs in Dak Lak, Vietnam grouped strongly (99 % support) within the zoonotic haplotype of *A. ceylanicum* within a subgroup (bootstrap value <50 %, not shown) comprising dog, cat and human isolates from Malaysia and Cambodia (Fig. 2). In rural communities, owned but free-roaming dogs defecate promiscuously and contaminate the environment with hookworm larvae. The high prevalence (67 %) of *A. ceylanicum* (and *A. caninum*) in these dogs coupled with poor standards of environmental hygiene, places co-residing humans at risk of acquiring zoonotic ancylostomiosis [29]. This study also advocates a molecular approach be adopted for future human parasite surveys in Vietnam. *A. ceylanicum* as the second most common hookworm species infecting humans in the region [9, 29–31], with natural infections reported in almost all geographical areas in which the hookworm is known to be endemic in dogs and cats [1]. It is therefore likely that this hookworm may be present in Vietnam, but overlooked in human parasite surveys.

## Conclusions

The results of this study challenge previous reports of the presence of *A. braziliense* and *U. stenocephala* in dogs in Vietnam. Instead, we provide strong evidence that the emerging parasite *A. ceylanicum* is the predominant hookworm of dogs in the region. This important finding begets that human parasite surveys in Vietnam do not overlook the presence and impact of this zoonosis and adopt a One Health approach for its control.

## Abbreviations

PCR: Real-time polymerase chain reaction
RFLP: Restriction Fragment Length Polymorphism
S.G.: Specific Gravity
COX-1: Cytochrome oxidase – 1
w/v: weight/volume
